# Chemical Characterization, Lipid Profile, and Volatile Compounds in *Chlorella* sp. and *Spirulina platensis*: A Promising Feedstock for Various Applications

**DOI:** 10.3390/molecules30071499

**Published:** 2025-03-27

**Authors:** Lacrimioara Senila, Eniko Kovacs, Cecilia Roman

**Affiliations:** Research Institute for Analytical Instrumentation Subsidiary, National Institute for Research and Development of Optoelectronics Bucharest INOE 2000, 67 Donath Street, 400293 Cluj-Napoca, Romania; lacri.senila@icia.ro

**Keywords:** microalgae, lipids, volatiles, nutritional indices

## Abstract

Microalgae are among the most promising feedstocks for a wide range of applications due to their ease of cultivation, rapid growth rate, and ability to accumulate significant amounts of lipids and other valuable compounds. In the current study, two microalgae species, *Chlorella* sp. and *Spirulina platensis*, were studied regarding chemical composition, lipid extraction by ultrasound-assisted solvent extraction, and volatile compounds analysis. The optimization of the lipid extraction process was investigated with respect to the influence of different process parameters. The highest lipid content was found in *Chlorella* sp., which was more than twice as high compared to *Spirulina platensis.* Both microalgae contain saturated fatty acids (SFAs), monounsaturated fatty acids (MUFAs), and polyunsaturated fatty acids (PUFAs). *Spirulina platensis* contains high palmitic acid (42.9%) and linolenic acid (22.5%), and is low in MUFA content (8.5%), whereas *Chlorella* sp. contains high oleic (21.9%), linoleic (25.3%), and α-Linolenic acid (10.2%). Based on the fatty acids profile, nutritional lipid indices were calculated. Regarding the volatile content, *Spirulina platensis* contains amines, aldehydes, alcohols, ketones, and hydrocarbons, whereas *Chlorella* sp. contains hydrocarbons, heterocycle, aldehydes, thiocyanates, and esters which give the odor profile.

## 1. Introduction

The global environmental, social, and economic concerns, mainly due to greenhouse gas emissions, fossil fuel depletion, global population growth, and energy insecurity are driving towards the use of renewable resources and the production of environmentally sustainable alternative products. In this regard, microalgae have the potential to serve as a feedstock for a variety of applications, including the production of biofuels, food, feed, cosmetics, and even healthcare products. Microalgae have attracted considerable attention due to their high lipid content, rapid growth rates, and ability to be cultivated without competing with food crops [1,2,3]. Algae, similar to corn, soybeans, sugar cane, wood, and other plants, use photosynthesis to convert solar energy into chemical energy. They then store this energy in the form of oil, carbohydrates, and proteins.

Microalgae are defined as photosynthetic aquatic organisms that are typically less than 0.01 mm in diameter. They are distinguished by their ability to undergo rapid and continuous growth [4], considerably faster than terrestrial species [5,6]. The mass of microalgae typically doubles daily. The cultivation of microalgae is a process that requires the presence of certain raw materials, namely sunlight, water, carbon dioxide (CO_2_), and nutrients (particularly phosphorus (P) and nitrogen (N)) [7]. The biodiesel production process from microalgae contains the following stages: algae cultivation, harvesting, lipid extraction, and transesterification process [8]. Depending on their size, there are two types of algae: microalgae and macroalgae. Microalgae are divided into four classes: diatom algae (*Bacillariophyceae*), green algae (*Chlorophyceae*), blue-green algae (*Cyanophyceae*), and yellow algae (*Chrysophyceae*) [9]. Diatom algae are the most widespread type of algae on Earth. It is estimated that the number of these species exceeds 100 thousand. The major components found in microalgae are lipids, carbohydrates, proteins, and nucleic acid. The concentration of lipids depends on algae types and can vary between 20 and 50% [10].

According to the literature, the lipid and fatty acid content of microalgae biomass varies depending on the species and cultivation conditions, and can yield up to 40–50% oil by weight. The species *Chlorella* has attracted research due to its potential application in various industries, given its wide availability and ease of cultivation under laboratory conditions [11]. Certain species of microalgae are employed in the context of wastewater treatment, *Chlorella* sp. being a notable example. These organisms have been observed to reduce biological oxygen demand, chemical oxygen demand, and phosphorous, while concurrently increasing lipid production [12]. Some studies investigated the potential of different microalgae species (*Chlorella*, *Desmodesmus*, *Paracoccus Chlamydomonas*) and bacteria consortium in wastewater treatment [13,14]. Wastewater bioremediation integrates the cultivation of microalgae with environmental remediation, reduces the need for costly synthetic nutrients, and promotes rapid algal growth, due to the high nutrient content. However, to ensure biomass safety for specific applications, such as food or feed production, it requires additional processing [15,16]. In a study reported by Esipovich et al. (2025), *Chlorella* biomass was used as feedstock for biofuel and value-added chemicals. *Chlorella* and *Spirulina* are considered important food sources due to their remarkable compounds [17,18]. *Chlorella protothecoides*, *Chlorella sorokiniana*, and *Spirulina platensis* are among the few microalgae used in food products that have been approved by the US Food and Drug Administration as Generally Recognized as Safe (GRAS) [19,20]. *Chlorella* sp. is characterized by a simple structure, rapid growth rates, and the capacity to double its biomass within a few hours, its growth not being limited by season, and its high degree of adaptability [21].

There are around 30 species of *Chlorella*, with *Chlorella vulgaris* being the most commercialized. Gouveia and Oliveira (2008) [22] studied six species of microalgae for biodiesel production: *C. vulgaris*, *Spirulina maxima*, *Nannochloropsis* sp., *Neochloris oleoabundans*, *Scenedesmus obliquus*, and *Dunaliella tertiolecta*. Among the species tested, *N. oleoabundans* (freshwater microalgae) and *Nannochloropsis* were found to be the most suitable for biodiesel production due to their high oil content (29% and 28.7%, respectively) [23]. James et al. (2025), in a study on marine microalgae as a feedstock for biofuel production, presented the methods used for the extraction of lipids in order to maximize lipid yields, and the process of transesterification, on a large-scale microalgae cultivation [24].

Spirulina is a nutrient-rich superfood known for its numerous health benefits, including anti-inflammatory effects and antioxidant properties. It contains both essential and non-essential amino acids, fatty acids, and powerful antioxidants [25]. Additionally, spirulina is an excellent source of protein, with amino acids comprising approximately 38–48.7% of its total protein content. *Spirulina* is also a great dietary supplement as it contains nutrients, vitamins, minerals, and essential fatty acids like omega-6 and omega-3. Also, Spirulina contains antioxidants like beta-carotene, phycocyanin, and various phenolic compounds [26]. Spain, France, and Ireland are the primary producers of Spirulina. Within the European Union, 222 producers were identified [27]. However, despite the superior overall nutritional properties of *Spirulina* and microalgae in general, the sensorial properties have a significant impact on consumer perception [28]. The distinctive odor and taste are associated with the presence of volatile compounds, among which the aldehydes, ketones, and alcohols groups are the most characteristic [29]. Aldehydes provide grassy, oily, or fruity odors; ketones provide green or mushroom odors; and alcohols provide fruity odors [28,30,31]. The investigation of volatile compounds responsible for the flavor is important, as it facilitates the development of methods that can lead to the improvement of microalgae-derived food products [28,29].

Lipids can be divided in the following classes: fatty acyls, glycerolipids (TAG or trigcerols), sterol, prenols, polyketides, glycerophospholipids, sphingolipids, and saccharolipids [32]. Depending on their structure, lipids are divided into polar and non-polar lipids. The non-polar lipids include polyketides, prenol lipids, glycerolipids, and fatty acids, whereas the polar lipids include saccharolipids, sphingolipids, glycerophospholipids and terol lipids. Fatty acids (FAs), glycerolipids, phospholipids, sphingolipids, and glycolipids are used in the production of biodiesel. Among the lipids, glycerolipids are the most preferred for biodiesel production. According to the number of carbon atoms, FAs are classified into FAs containing 14–20 carbon atoms used for the production of biodiesel and polyunsaturated fatty acids (PUFAs) with more than 20 carbon atoms. Furthermore, according to the presence or absence of double bonds, fatty acids are classified into saturated (SFAs—no double bonds), monounsaturated (MUFAs—with one double bond) and polyunsaturated fatty acids (PUFAs—with two or up to six double bonds).

Advances in chemical methods for lipid extraction encompass supercritical carbon dioxide, enzymes, engineered nanoparticles, bio-based solvents, ionic liquids, switchable solvents, and deep eutectic solvents [33]. The conventional chemical method for lipid extraction involves the use of solvents. The extraction of oil from microalgae depends on the solvent used. The most commonly used solvents for lipid extraction are chloroform, methanol, hexane, ethanol, petroleum ether, and cyclohexane. Various mixtures of solvents have been used to improve extraction, such as chloroform–methanol or hexane–isopropanol. The extraction of non-polar lipids (hydrophobic character) is most effectively conducted using a non-polar solvent (chloroform and hexane), whereas polar solvents are recommended for the extraction of polar lipids (amphiphilic character). Reaction temperature, particle size, and solvent/solid ratio also affect the lipid extraction. The most appropriate solvents for the extraction of lipids from microalgae are those with a low boiling point, high specificity, insolubility in water, volatility, and non-toxic properties. The lipid extraction methods employed for microalgae are the Folck extraction, Bligh and Dyer, Soxhlet, supercritical fluid extraction, microwave-assisted extraction, and ultrasound-assisted extraction. The extracted lipids contain compounds that are not suitable for transesterification, such as chlorophyll and magnesium porphyrin complex [34]. The end-to-end purification of the oil is an essential procedure.

The objectives of this study were the following: (i) to extract lipids from two microalgae species (*Spirulina platensis* and *Chlorella* sp.) using various solvent mixtures, (ii) to optimize the lipid extraction efficiency by investigating the effects of solvent types, temperature, and solvent-to-solid ratio, (iii) to determine the fatty acids composition (SFAs, MUFAs, PUFAs, omega-6, omega-3) of lipid separated from both microalgae, (iv) lipid nutritional quality, and (v) volatile content in order to establish the flavor and compound that contribute to lipid oxidation.

## 2. Results and Discussion

### 2.1. Chemical Composition of the Raw Material

The characteristics of the microalgae studied are given in Table 1.

The moisture content was below 2% for both species. The protein content of *Spirulina platensis* biomass was calculated to be 65.6%, whereas *Chlorella* sp. contained 59.3% protein. The content of nitrogen in *Spirulina platensis* is higher, suggesting a significant protein content. The lipid content of *Chlorella* sp. was found to be 17.4%, while *Spirulina platensis* exhibited a lipid content of 7.2%. In a related study, Ambrozova et al. (2014) reported a lipid content of 18.02% in *Chlorella kessleri* and 10.23% in *Spirulina platensis* [35]. The biomass’ content of protein and ash was higher in *Spirulina platensis* than in *Chlorella*, according to Ladjal-Ettoumi et al. (2024) [26]. In a study reported by Baba et al. (2016), a cellulose content of 25% was found in the *Nannochloropsis gaditana* microalgae species, a type of microalgae employed for biodiesel production [36]. Similarly, Kavitha et al. (2021) reported a cellulose content of 33% along with an 18.2% hemicellulose content for the same microalgae species, when used as a raw material for ethanol production [37]. The studied microalgae do not contain lignin, but have a low hemicellulose content. According to Silva et al. (2025), microalgae polysaccharides are heteropolymers composed of galactose, xylose, glucose, arabinose, fucose, mannose, rhamnose, and glucuronic and galacturonic acids. Generally, the content of cellulose is between 1.7 and 24.2%, depending on the species [38]. Demirbaş (2008) reported 51–58% protein, 12–17% carbohydrates, and 14–22% lipids for *Chlorella vulgaris*, and 57% protein, 26% carbohydrates, and 2% lipids for *Chlorella pyrenoidosa*. The reported contents in *Spirulina platensis* ranged between 46 and 63% for protein, 8–14% for carbohydrates and 4–9% for lipids, while for *Spirulina maxima*, these ranged between 60 and 71% for protein, 13–16% for carbohydrates, and 6–7% for lipids [39].

### 2.2. Volatile Compounds from Microalgae

A total of 43 volatile compounds were identified in *Chlorella* sp. and *Spirulina platensis* microalgae (Table 2). The volatile compounds were identified by comparing the retention indices (RI) and the mass spectra obtained from the NIST MS Search 2.3 database. The identified volatile organic compounds are classified in twelve compound groups: amines, aldehydes, alcohols, ketones, hydrocarbon, heterocycle, acids, ethers, furans, nitrogen compounds, sulfur compounds, and esters (Table 2). The compounds found in high quantities in *Chlorella* are 2-pentyl-furan (16.33%), N-methylaziridine (13.13%), hexanal (9.48%), propyl cyanate (8.57%), 9-aza-10-boradecalin (7.82%), methyl sulfocyanate (7.25%), and 4,4-dimethylcyclopentene (7.24%). The compounds found in high amounts in *Spirulina platensis* are hexanal (19.76%), N-methyl-4-pyridinamine (15.71%), cis-(3,3,5)-trimethylcyclohexanol (12.43%), 2,2,6-trimethylcyclohexanone (11.32%), and 1-oxaspiro[2.5]oct-5-ene, 8,8-dimethyl-4-methylene (7.47%). Heterocycles have the highest relative abundance (18.52%) in *Chlorella* sp., followed by furans (18.09%), esters (17.03%), nitrogen compounds (14.4%), and aldehydes (10.68%). In *Spirulina platensis*, aldehydes are predominant (24.86%), followed by alcohols (21.98%) and ketones (14.24%). Also, a high quantity of hydrocarbon (13.23%) was found in *Spirulina platensis*.

The heterocyclic compounds identified are 6-methyl-2-azabicyclo[2.2.0]hex-5-en-3-one, 1,2,3,6-tetrahydropyridine, azetidine, 5-formamidopyrimidine, 9-aza-10-boradecalin, 1-oxaspiro[2.5]oct-5-ene, 8,8-dimethyl-4-methylene, and tetrahydropyridine.

The fruity, sweet, and caramel aroma is given by the furan compounds (furan, 2-pentyl-furan). 2-Pentyl-furan appears in both *Spirulina platensis* and *Chlorella* sp., suggesting a shared metabolic pathway. This compound gives grassy aromas and contributes to lipid oxidation. It was suggested that 2-pentyl-furan is formed upon the lipoxygenase-catalyzed oxidation of linoleic acid [40]. The pyridine compounds were introduced into heterocycle compounds. The majority of these compounds are related to the content of sugars and amino acids from microalgae. Sulfur compounds are present in low quantities and may contribute to the pungent, acrid flavor and are formed from the possible presence of sulfurous amino acids present in microalgae (ex. methionine and cysteine). During algal metabolism some compounds, such as amine and aziridine, are produced during the degradation of amino acids. Compounds with sulfur or nitrogen are produced during algae stress (in the presence of nitrogen). Furan is formed during the oxidation or thermal degradation of lipids [28].

**Figure 1:**
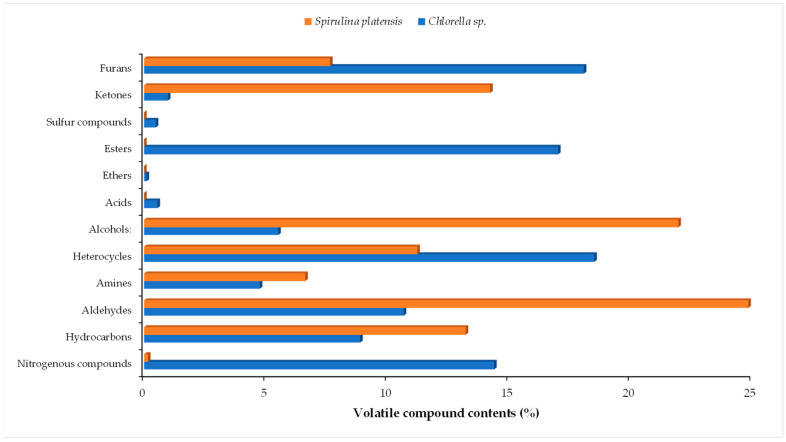
Classification of volatile organic compounds and their average relative abundance (%) identified in microalgae species.

Figure 1 shows the classification of volatile compounds by group classes (percentage), for both microalgae sp. The abundance of alcohols is 21.98% in *Spirulina platensis* and 5.52% in *Chlorella* sp., contributing to their flavors. [41]. 3,5-Dimethylcyclohexanol and *cis*-(3,3,5)-trimethylcyclohexanol contribute to the fruity, green note, but their levels are not significantly high. According to Urlass et al. (2023), volatiles in the alcohols group are produced by the degradation of secondary hydroxyperoxide fatty acids. Other hypotheses assume that they originate in the glycolysis reaction of amino acids through the Ehrlich pathway or in the degradation of omega-3 PUFAs [42].

The abundance of amines among volatile organic compounds is 6.63% in *Spirulina platensis* and 4.76% in *Chlorella* sp. Both microalgae have high protein content, including all essential amino acids. In addition, microalgae also contain vitamins, antioxidants, and amino acids. The specific amines found in *Spirulina platensis* include N-methylallylamine, 2-propen-1-amine, N-methyl-4-pyridinamine, ethylmethylamine, N,N-2-trimethylpyridin-4-amine, whereas in *Chlorella* the following amines are identified: 2-propyn-1-amine, 3,4-pyridinediamine, and 1H-tetrazol-5-amine. 2-Propen-1-amine is formed by decarboxilation of amino acids from microalgae. Some amino acids, such as histidine and glutamate, can form allylamine. The existing literature on volatile compounds from microalgae is extremely limited. In their study, Villaró et al. (2023) reported on the volatiles of *Spirulina platensis* harvested in both freshwater and seawater, finding that some volatiles were present in different quantities [43].

The identification of other volatiles in seawater microalgae and freshwater microalgae can be attributed to various factors, including salinity, microbial communities, nutrient types, oxygenation levels, and the presence of light [43].

Aldehydes are found in *Chlorella* sp. with an abundance of 10.68% and in *Spirulina platensis* with an abundance of 24.86%. These compounds contribute to the flavor profile of microalgae. Zhao et al. (2024) reported a flavor profile of four microalgae: *Spirulina platensis*, *Chlorella pyrenoidosa*, *Chlamydomonas reinhardtii*, and *Haematococcus pluvialis,* identifying ketones, aldehydes, esters, alcohols, nitrogen compounds, organic acids, furans, phenols, sulfur compounds, and hydrocarbons [44]. Aldehydes are obtained from the oxidative degradation of unsaturated fatty acids or triglycerides. Hexanal is a volatile compound identified in both microalgae in high concentrations. It is produced by the oxidation of PUFAs and gives a green, grassy, sweet, and citrus aroma. The aldehydes 2-methyl-2-butenal and 4-methylbenzaldehyde were found only in *Chlorella* sp. and give a strong, fruity, and sweet astringent flavor and are derived from oxidation of fatty acids. Benzaldehydes have been identified in other algae, such as *Rhodomonas* sp., *Tetraselmis* sp., *C. vulgaris*, *B. braunii*, *U. pertusa*, and *N. oculate* [42].

The origin of volatile compounds found in *Chlorella* sp. and *Spirulina platensis* can be attributed to various sources, such as lipids, proteins, and hydrocarbons metabolism. The presence of various volatile compounds groups impacts the odor profile, which can vary from pleasant (fruity, citric) to unpleasant (sulfurous, rancid). Braga-Souto et al. (2024) investigated *Spirulina* and the compounds involved in its distinctive odor, taste, and flavor profile, and indicated that the profile is dependent on the predominant compounds [45]. The different ionone derivates from the ketone group are considered responsible for the fruity, flowery and woody odor, aldehydes provide a grassy odor, alcohols can give a herbal, soapy odor, while pyrazines can contribute to nutty, green, or fatty odor [28]. In a study conducted by Zhao et al. (2024), the presence of ketones was the contributing factor to the pleasant floral and fruity odor [44]. Volatile compounds have been shown to significantly influence lipid oxidation in microalgae, and reactive ketones, for example, are a result of lipid oxidation [46].

The volatile organic compounds identified in *Chlorella* sp. and *Spirulina platensis* are listed in Table 2.

These two types of microalgae can be used to extract important compounds used in different applications, including medicine, food, biofuel, bioplastics, biochemical and industrial applications, dietary bioactive compounds, and nanoparticles biosynthesis [47,48,49].

This study demonstrated that *Chlorella* sp. and *Spirulina platensis* function as reservoirs of volatile organic compounds. The study serves to broaden the existing knowledge base concerning chemical compounds and odor profiles specific to each group. A significant proportion of the identified compounds can be extracted and utilized in various applications, including, but not limited to, the food, pharmaceutical, and cosmetic industries [18,50].

### 2.3. Extraction of Lipids from Microalgae

In the present study, ultrasound-assisted solvent extraction was applied for the separation of oil from microalgae. The generation of ultrasonic waves by a reactor leads to the formation of bubbles in the solvent, which subsequently break at the level adjacent to the cell walls, thereby generating shock waves and jets of liquid that result in the rupture of the cells and the subsequent release of their contents. According to Khoo et al. (2023), ultrasound-assisted extraction aims to disrupt the cytoplasm of the cells and release lipid molecules [33]. In this study, ultrasound-assisted extraction with various solvents was tested in order to find the optimal conditions for the highest oil yield. In this sense, three types of parameters were evaluated: influence of solvent mixture, temperature, and solid-to-solvent ratio.

#### 2.3.1. Effects of Solvent Mixtures

**Figure 2:**
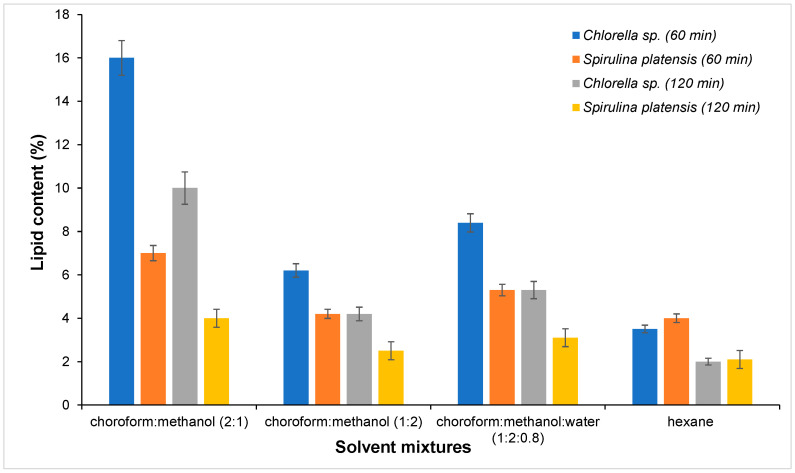
Effects of various solvent mixtures on lipid extraction from *Chlorella* sp. and *Spirulina platensis* biomass (microalgal biomass: 4 g, ultrasound, extraction time: 60 min, solvent to solid ratio: 20:1, temperature: 25 °C).

The following solvent mixtures were tested: chloroform–methanol (2:1), chloroform–methanol (1:2), chloroform–methanol–water (1:2:0.8), and hexane. It is noteworthy that all experiments employed a 20:1 solvent-to-solid ratio and utilized ultrasound for a duration of 60 min. The lipid content obtained after extraction with different solvent mixtures from *Chlorella* sp. and *Spirulina platensis* is presented in Figure 2. The highest lipid yield was obtained using the chloroform–methanol ratio of 2:1, with *Chlorella* sp. exhibiting approximately 16% lipid content in comparison to approximately 7% in *Spirulina platensis*. These results are in accordance with the existing literature on the subject [33]. It was found that the most effective solvent mixture for lipid extraction in microalgae was a combination of chloroform and methanol in a 2:1 ratio. However, it was observed that utilizing a chloroform–methanol ratio of 1:2 led to a decline in lipid extraction across both species. Conversely, the addition of water resulted in a marginal enhancement in lipid extraction in *Chlorella* sp.

Hexane, on the other hand, was found to be the least effective extraction agent, due to its capacity to extract only neutral lipids (triglycerides). The presence of water in solvent mixtures improves the lipid extraction from *Chlorella* sp. The increase in the lipid extraction from *Chlorella* sp. with water addition could be due to the differences in their cell composition and structure. Due to low carbohydrate content and high lipid content comparative with *Spirulina*, the addition of water may help to disrupt hydrogen bond into sugars, facilitating lipid release. The water molecules form hydrogen bond interaction between hydrogen bond acceptor and hydrogen bond donor (amides, amines, and alcohols). According to Ren et al. (2017), the addition of water in lipid extraction from *Chlorella protothecoides* increases lipid extraction by releasing intracellular lipids and improves the lipid extraction yield [51].

The extraction time was increased from 60 to 120 min, but a decrease in lipid yield was observed for both species (Figure 2).

#### 2.3.2. Effects of Extraction Temperature

The present study investigated the influence of temperature on lipid extraction. The ultrasound temperature was set to 25, 40, 50, and 60 °C for each solvent tested. The extraction time was set to 60 min, and the solid to solvent ratio was set to 1:20. The optimal temperature for lipid extraction was determined to be 25 °C for both microalgae species. It is demonstrated that an increase in the extraction temperature results in a corresponding decrease in the extraction yield for both algae species. This is due to the decreasing density of the fluids [52]. According to Deng et al. (2022), high temperature and long extraction time lead to lipid oxidation. In addition, ultrasound could improve the extraction efficiency and lipid quality [53].

Ultrasound-assisted extraction can improve lipid extraction by disrupting the cells. The waves (>20 kHz) create cavitation bubbles that break up the cell walls of microalgae and release intracellular lipids more easily. According to the literature, ultrasound extraction improves lipid extraction compared to conventional methods, reduces the use of solvents, and shortens the extraction time. The solvents used, their viscosity, and surface tension, influence the lipid extraction [54,55].

**Figure 3:**
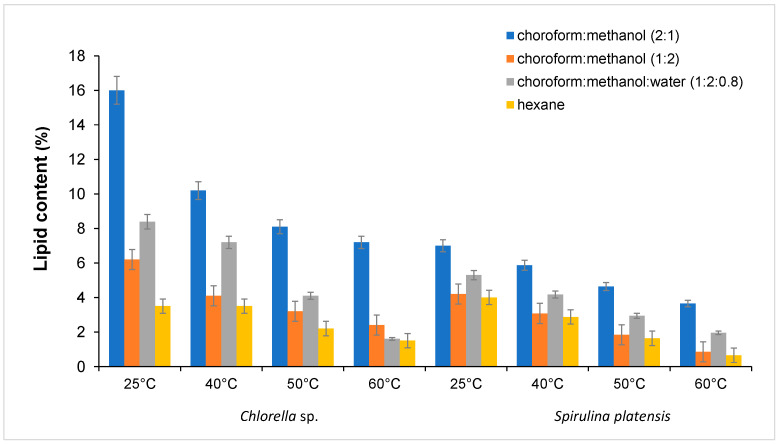
The influence of temperature on the extraction yield (microalgal biomass: 4 g, ultrasound, extraction time: 60 min).

Furthermore, the method has been shown to have a beneficial effect on the composition of lipids, as well as on their antioxidant and antimicrobial properties [53]. In the study conducted by Krishnamoorthy et al. (2023), it was found that ultrasonic cavitation induced within cells led to the breakdown of the intracellular components. In the same study, the extraction of lipids from *Chlorella vulgaris* and *Nannochloropsis oculata* was investigated in the absence of ultrasound, yielding a result of only 4.64% [56]. *Chlorella* sp. had a higher lipid content (15.0%) compared with *Spirulina platensis*, which recommended it as raw material for biodiesel production. The effects of temperature on the extraction yield are depicted in Figure 3.

#### 2.3.3. Effects of Solid-to-Solvent Ratio

The solvent-to-solid ratio is known to have an important impact on microalgal lipid extraction. Therefore, its optimal value must be determined in order to verify the economic feasibility. Different solid-to-solvent ratios have been investigated, (1:10, 1:20, 1:30, and 1:50) for 60 min at 25 °C. The extraction yield increases with the increase in the solid-to-solvent ratio from 1.3 (1:10) to 6.5% (1:50) for *Spirulina platensis* and from 7.2 (1:10) to 15.6% (1:50) for *Chlorella* sp. The optimal parameters for lipids extraction over 60 min with chloroform–methanol (2:1) were as follows: a solid-to-solvent ratio of 1:20 for *Spirulina platensis* and a solid-to-solvent ratio of 1:50 for *Chlorella* sp. The optimal solvent mixture was chloroform–methanol (2:1) for both species. In order to improve the microalgal lipid extraction from *Spirulina platensis* biomass, increasing the solid-to-solvent ratio beyond 1:20 had little effect. Contrarily, for *Chlorella* sp., the trend continued to slowly increase up to 1:50 solid-to-solvent ratio. A higher solvent volume increases the concentration gradient and improves the oil yield, but it also increases the operation cost. Methanol was added as a co-solvent to improve the oil yield, and its presence had a positive effect on the extraction yield. Other studies also reported the use of a co-solvent in the extraction phase. Pre-treatment methods, such as ultrasonication, enhance lipid release by breaking down the resistance of the cell walls [56].

### 2.4. Fatty Acids Composition in Oil Extracted from Microalgae

Fatty acid methyl ester (FAME) composition of *Chlorella* sp. and *Spirulina platensis* is presented in Table 3.

Lipids consist of FAs which are classified by the presence or absence of double bonds: saturated fatty acids (SFAs—without double bonds), monounsaturated fatty acids (MUFAs—with one double bond), and polyunsaturated fatty acids (PUFAs—with two or up to six double bonds). The saturated (SFAs), monounsaturated (MUFAs), and polyunsaturated fatty acids (PUFAs) were identified in *Chlorella* and *Spirulina platensis* oil extracts. The main FAMES found by GC-FID were palmitic acid (C16:0), oleic acid (C18:1), and linoleic acid (C18:0). The SFAs classes include: capric acid (C10:0), undecanoic acid (C11:0), myristic acid (C14:0), palmitic acid (C16:0), stearic acid (C18:0), arachidic acid (C20:0), and heneicosylic acid (C21:0). Palmitic acid (C16:0) was the most abundant SFA in almost every sample. *Spirulina platensis* has a higher total SFA content (50.1%) than *Chlorella* sp. (35.9%). PUFAs were found to be around 41% in both species. PUFAs contain both omega-6 and omega-3. This finding indicates that algae generally produce a high amount of polyunsaturated fatty acids, which may pose a stability issue due to the correlation between high polyunsaturated fatty acids content and reduced oxidation stability of the biodiesel obtained [33]. *Chlorella* sp. has a higher omega-3 content, while *Spirulina platensis* has a higher omega-6 content. The group of omega-6 (n-6) is represented by linoleic acid (C18:2, n-6), γ-linolenic acid (C18:3, n-6), and *cis*-11,14-Eicosadienoic acid (C20:2 (n-6). *Chlorella* sp. is rich in MUFAs, whereas *Spirulina platensis* is rich in PUFAs, especially γ-linolenic acid. For instance, eicosapentaenoic acid (EPA, C20: 5n-3; five double bonds) and docosahexaenoic acid (DHA, C22: 6n-3; six double bonds) are prevalent in algal oils [39]. *Chlorella* sp. has 0.42% DHA of the total lipids. Fatty acids and fatty acid methyl esters of fatty acids (FAMEs) with four or more double bonds are susceptible to oxidation during storage, which reduces their acceptability for use as biodiesel. According to Silva et al. (2025), EPA and DHA have several protective benefits for human health, including risk of neurological disorders, brain inflammation, cardiovascular diseases, and cancer [38].

However, polyunsaturated fatty acids have much lower melting points than monounsaturated or saturated fatty acids, so algae-based biodiesel should perform much better at low temperatures compared to many other types of biodiesels. Due to the fact that certain strains of algae produce high amounts of polyunsaturated fatty acids, which are generally found in fish oils, they represent important sources for obtaining polyunsaturated fatty acids (omega-3 and omega-6), which are essential in human nutrition. These additional products significantly increase the marketability and overall economics of algae production [57]. The lipids obtained from microalgae can be used for biodiesel production by transesterification or in the food and medicine industries due to the high content of PUFAs. The consumption of food with high PUFA content reduces the risk of cardiovascular disease. In aquaculture and animal feed, PUFAs are used as adjuvant in meat products. Therefore, an analysis is needed in order to identify the volatile compounds that contribute to their flavor. PUFAs also have cosmetic applications due to their biological activities [44].

The FFAs content of the oil obtained from *Spirulina platensis* is 0.52 mg KOH/g, and from *Chlorella* sp. is 1.41 mg KOH/g.

The unique chemical composition and properties of *Chlorella* and *Spirulina* render them suitable for various applications. However, the viability of these applications depends on a number of factors, such as market demand, production costs, and technological advancements [58]. The high lipid content is an important factor in the production of biodiesel, as well as in the food and cosmetics industries, enhancing the economic viability through the multiple possible uses of these microalgae [59]. However, the economic feasibility and scalability is limited by the high production costs [60,61].

### 2.5. Lipid Quality Indices

Table 4 presents the calculation of the lipid nutritional indices from the FAME for Chlorella sp. and Spirulina platensis.

According to Chen and Liu (2020), PUFA/SFA is an index used for the evaluation of the impact on cardiovascular disease. PUFAs have been demonstrated to have a beneficial effect on health, whereas the content of SFAs has been shown to have a positive impact on high cholesterol levels [62]. The content of omega-6 is higher in *Spirulina platensis* than in *Chlorella* sp., indicating a higher amount of linoleic acid, whereas omega-3 is higher in *Chlorella* sp., indicating a better cardiovascular benefit in case of consumption.

The *Chlorella* sp. sample exhibited a higher PUFA/SFA ratio (1.14), indicative of a substantial polyunsaturated fatty acid content, which is known to promote cardiovascular health. The saturated fatty acids (SFAs) that contribute to increased cholesterol levels are C10:0, C11:0, C12:0, C14:0, C15:0, C16:0, C17:0, C18:0, and C20:0. In accordance with Chen and Liu’s (2020) findings [62], the PUFA/SFA content levels of various foodstuffs are as follows: 0.42–2.12% for seaweed, 0.11–2.042% for meat, 0.50–1.62% for fish, 0.20–2.10% for shellfish, and 0.02–0.175% for dietary products. The MUFA/SFA ratio is 0.64 for *Chlorella* sp. and 0.17 for *Spirulina platensis*. The low quantities of MUFAs identified in *Spirulina platensis*, which predominantly contains SFAs, make it less beneficial in terms of cardiovascular health. The index of atherogenicity had a lower value for *Chlorella* sp. The AI index is indicative of the relationship between the sum of SFAs and UFAs. Lower values are preferable in order to reduce the risk of atherosclerosis. SFAs takes into account C14 and C16 fatty acids. The high content of PUFAs in microalgae contributes to their high UFA content.

The thrombogenicity index (TI) is 0.22 for *Chlorella* sp. and 0.28 for *Spirulina platensis*. A lower TI is better, and the results suggested that the consumption of both microalgae can reduce the risk of blood clot formation. TI is a ratio between pro-thrombogenic fatty acids (SFAs) and anti-thrombogenic fatty acids (MUFAs and PUFAs). Tavakoli et al. (2025) reported the following lipid indices for *Spirulina maxima*: a nutritive value index (NVI) of −0.06, an atherogenicity index (AI) of 0.28, a thrombogenicity index (TI) of 0.14, a hypocholesterolemic/hypercholesterolemic ratio (h/H) of 1.94, a health-promoting index (PI) of 158.89, and a linoleic acid/linolenic acid ratio of 0.36 [25]. The low AI and TI levels observed in both microalgae species suggest that these microalgae contain healthy lipids, and their consumption may have a beneficial effect on cardiovascular health. In the context of dietary choices, *Spirulina platensis* is regarded as a preferable food alternative, while *Chlorella* sp. is identified as a potential source for biodiesel production. The hypocholesterolemic/hypercholesterolemic ratio (h/H) is 2.3 for *Chlorella* sp. and 1.0 for *Spirulina platensis*. This index is the inverse of the AI and is defined as the ratio between the sum of unsaturated fatty acids (linoleic acid, linolenic acid, and oleic acid) and saturated fatty acids (myristic, stearic, and palmitic). *Spirulina platensis* has a higher SFA content compared to *Chlorella* sp. but a lower MUFA content.

The remarkable omega-6/omega-3 (13.0) report calculated in *Spirulina platensis* indicates high quantities of omega-6 and low quantities of omega-3.

The EPA + DHA content in *Spirulina platensis* (1.16) is higher than in *Chlorella* sp. (0.44), making it a better source of long-chain omega-6, which are important for brain and heart health. According to Zhou (2022), the microalgae with high contents of DHA and EPA have important physiological functions, including antibacterial, anti-inflammatory, and immune-regulating effects [63]. The same study showed that *Chlorella* has anti-inflammatory, anti-diabetic, and cardiovascular health effects, while *Spirulina platensis* has anti-diabetic and cardiovascular effects due to its PUFA extract, bioactive molecules generated by the oxidation of fatty acids, and DHA supplementation.

## 3. Materials and Methods

### 3.1. Chemicals and Raw Material

*Spirulina platensis* and *Chlorella* sp. were procured as biomass powder from an organic certified company (Cluj-Napoca, Romania). The chemicals used in the experiments—such as methanol (CH_3_OH), chloroform (CHCl_3_), isooctane (C_8_H_18_), potassium hydroxide (KOH), sodium hydrogen sulfate (NaHSO_4_), sodium sulphate (Na_2_SO_4_), potassium chloride (KCl), sodium hydroxide (NaOH), acetone (C_3_H_6_O), acetic acid (CH_3_COOH), benzoic acid (C_7_H_6_O_2_), sodium chloride (NaCl), hexane (C_6_H_14_), and silica gel—were purchased from Merck (Darmstadt, Germany). Sodium chlorite (NaClO_2_) (80%) was purchased from Alfa Aesar (Karlsruhe, Germany). An ultrapure water (Elga Veolia, High Wycombe, UK) system was used for sample preparation. A reference standard material Supelco 37 component FAME mix, CRM47885) was used for fatty acids identification and was purchased from Merck (Darmstadt, Germany).

### 3.2. Physico-Chemical Characterization of the Raw Material

#### 3.2.1. Cellulose and Hemicellulose Analysis

The cellulose content was determined by treating microalgae with sodium chlorite in an acetic acid solution (10%): 5 g of sample was treated with 5 g NaClO_2_ in 375 mL glacial acetic acid. The sample was mixed at 75 °C for 1 h (repeated for three times). The product was filtrated, washed with water and acetone, dried at 105 °C for 24 h in vacuo, and weighed. To determine the hemicellulose content, the dried solid was treated with 17.5% NaOH at 20 °C for 40 min, followed by the addition of 25 mL of water. The residue was filtrated, washed with 40 mL 10% glacial acetic acid and 1 L boiling water. The carbohydrates residue was filtrated, dried at 105 °C for 48 h in vacuo, and weighed.

#### 3.2.2. Moisture, Ash, Protein, and Nitrogen Content

The chemical characterization of microalgae samples analyzed the content of nitrogen, ash, and calorific value. The samples’ moisture was determined by drying them in a universal oven (UFE 400, Memmert, Schwabach, Germany) at 105 °C for 24 h. The protein content was calculated by multiplying the nitrogen (N) content by a nitrogen-to-protein conversion factor of 6.25. The nitrogen content was determined with a Flash EA 2000 CHNS/O analyzer (Thermo Fisher Scientific, Waltham, MA, USA). The ash content was determined by drying a quantity of microalgae in the oven at 550 °C for 5 h, and by quantifying the remaining solids.

#### 3.2.3. Calorific Value

The higher heating value (HHV) was determined by a 6200 Isoperibol calorimeter (Parr Instrument, Moline, IL, USA), calibrated by the combustion of certified benzoic acid. The dried microalgae were analyzed as given in the ISO 18125:2017 standard [64]. The weighed sample containing 0.4 g biomass and 0.6 g benzoic acid was placed in the sample holder of the bomb. The bomb was assembled, filled with oxygen for 30 s at a pressure of 400 psi, and placed in the calorimeter. The sample was burned under controlled conditions for 15 min (the temperature was recorded during combustion).

#### 3.2.4. Determination of Volatile Compounds from Microalgae

The volatile compounds were determined using a gas chromatograph coupled with a mass spectrometer (GC-MS) (6890N, Agilent Technologies, Santa Clara, CA, USA), equipped with a HP-5-MS capillary column (60 m length, 0.2 mm I.D., 0.25 µm film thickness) (Agilent Technologies, Santa Clara, CA, USA) and automated headspace G1888 (Agilent Technologies, Santa Clara, CA, USA). A quantity of 3 g of wet microalgae was transferred to a 20 mL headspace vial, with 3 g of NaCl added to assist in increasing the volatility of the compounds and to inhibit any enzymatic reactions. The temperature of the GC oven was set at 35 °C (held for 1 min), increased to 100 °C (held for 1 min) at a rate of 5 °C/min, then to 150 °C (held for 3 min) at 7 °C/min, and finally to 250 °C (held for 1 min) at a rate of 10 °C/min. The transfer line temperature was set at 280 °C and the ion source temperature was set at 250 °C. The headspace parameters are stated as follows: oven temperature: 70 °C, vigorous agitation; injection loop temperature (1 mL): 80 °C; HS transfer line temperature: 90 °C; equilibration time: 30 min; pressurization time: 0.15 min; filling time: 0.5 min; and injection time: 0.5 min.

The volatile compounds were identified by the NIST mass spectrometry library (NIST 11), and the identification of the chemicals was determined by matching with NIST 11 at ≥70% matching factors. All measurements were conducted in triplicate, and data are presented as the mean ± standard deviation.

### 3.3. Extraction of Lipids from Samples

**Figure 4:**
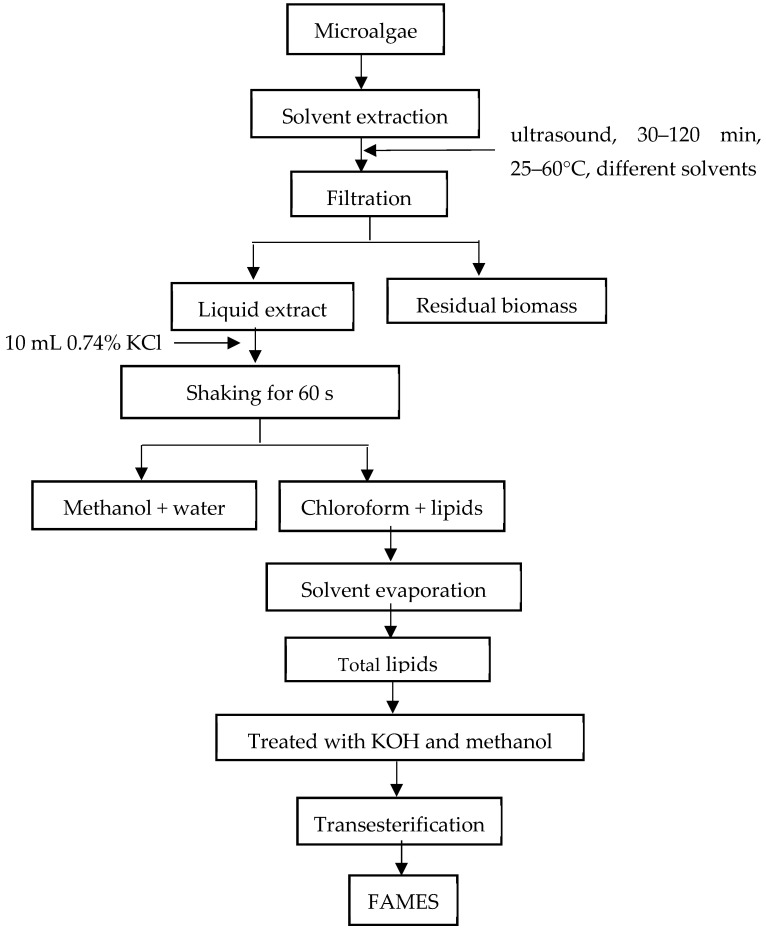
Process diagram of lipid extraction from *Chlorella* sp. and *Spirulina platensis* for chloroform–methanol (2:1), chloroform–methanol (1:2), and chloroform–methanol–water (1:2:0.8) solvents.

An amount of 5 g of dried microalgae was mixed with various solvent mixtures and extracted in an ultrasonic bath (Sonorex RK 512H, BANDELIN electronic GmbH & Co., KG, Berlin, Germany) for 30–120 min. The following solvents were used: chloroform–methanol (2:1), chloroform–methanol (1:2), chloroform–methanol–water (1:2:0.8), and hexane. The temperatures tested were 25–60 °C. After extraction, the remaining solids were stirred at 4000 rpm for 10 min, followed by filtration (using a Whatman No. 40 filter, Sigma-Aldrich, St. Louis, MO, USA) and separation into an organic phase. The organic phase was treated with 10 mL KCl (0.74%) for extraction and purification. The chloroform phase was separated, and the residual water was removed using Na_2_SO_4_. The solvent was removed using a rotary evaporator Laborota 4010 (Heidolph, Schwabach, Germany), and the oil was dried at 60 °C in an oven. The oil was purified in a silica gel/Al_2_O_3_ column to obtain pure oil. The lipid was determined gravimetrically as the ratio between the mass of extracted lipid and the mass of dried microalgae powder. In Figure 4 the process diagram of lipid extraction from *Chlorella* sp. and *Spirulina platensis* is presented. Lipid extraction with hexane was carried out in one stage, followed by evaporating the solvent without a second extraction with KCl.

#### 3.3.1. Fatty Acid Methyl Esters (FAMEs)

Approximately 1 g of the oil was dissolved in 4 mL of isooctane and then esterified with 200 µL of methanolic potassium hydroxide solution (CH_5_KO_2_, 2 mol/L). The mixture was vigorously shaken for several seconds. Subsequently, 1 g of sodium hydrogen sulfate (NaHSO_4_ H_2_O) was added, and the liquid fraction was filtered (using a Whatman No. 40 filter) before being directly injected into the gas chromatograph.

#### 3.3.2. Determination of FAMEs Content Using GC-FID

The FAMEs content in the microalgae oil samples was analyzed using a gas chromatograph with a flame ionization detector (GC-FID) (Agilent Technologies, 6890N, Santa Clara, CA, USA), and a ZB-WAX capillary column (30 m × 0.25 mm × 0.25 µm) (Agilent Technologies, Santa Clara, CA, USA). Helium was used as the carrier gas at a constant flow rate of 1 mL/min, with a split ratio of 1:20, and an injected volume of 1 µL. The temperature program for the GC oven consisted of three stages: 60 °C for 1 min, a ramp from 60 °C to 200 °C at 10 °C/min for 2 min, and a ramp from 200 °C to 220 °C at 5 °C/min for 20 min. Both the injector and detector temperatures were set at 250 °C to ensure the full vaporization of the sample and optimal detection sensitivity. The retention times of the sample FAMEs were compared to those of the Supelco FAME standard mixture (Sigma-Aldrich, St. Louis, MO, USA).

#### 3.3.3. Free Fatty Acid (FFA) Content from Extracted Oils

The FFA content was determined based on the acid value by dissolving the samples in a mixture of solvents (diethyl ether–ethanol, 1:1, *v*/*v*) and 2% phenolphthalein (in ethanol) as an indicator, then titrating with KOH (0.1 M in ethanol). The FFA was calculated with the Equation (1):

(1) $$FFA=\frac{V*56.1*C}{m}mg\;KOH/g$$

where *V* is the volume of KOH used for titration (mL), 56.1 is the molecular weight of KOH (mg/mmol), *C* is the concentration of KOH (m mol/mL), and m is the mass of the analyzed sample (g) [65].

### 3.4. Lipid Nutritional Indices

The fatty acids were used for the calculation of various lipids nutritional indices, such as SFAs, MUFAs, PUFAs, unsaturated fatty acids (UFAs), omega-6/omega-3 (ω6/ω3), thrombogenic index (TI), atherogenic index (AI), hypocholesterolemic/hypercholesterolemic ratio (h/H), health-promoting index (HPI), and nutritive value index (NVI), and they are presented in Table 5 [66].

### 3.5. Statistical Analysis

The statistical analysis was performed with the Tukey’s test (*p* = 0.05) using the Paired Comparison App (two-way ANOVA) by the Origin software (version 2020b, OriginLab, Northampton, MA, USA). The different letters indicate statistically significant differences at a level of *p* < 0.05.

## 4. Conclusions

The study investigated the chemical composition, lipid profile, and volatile compounds of two microalgae species, *Spirulina platensis* and *Chlorella* sp., in order to assess their suitability for diverse applications. It focused on methods for optimizing lipid extraction and characterization techniques for the two microalgae species. Additionally, it addressed the lipids nutritional quality.

*Spirulina platensis* had a higher carbohydrates content compared to *Chlorella* sp. The lipid content of *Chlorella* sp. was 17.4%, while *Spirulina platensis* exhibited a lipid content of 7.2%. Ultrasound-assisted solvent extraction was applied for the separation of oil from microalgae. The highest lipid yield was obtained using a chloroform–methanol ratio of 2:1, with *Chlorella* sp. exhibiting a higher lipid content than *Spirulina platensis*. The presence of water in solvent mixtures improved the lipid extraction from *Chlorella* sp. for chloroform–methanol (1:2) solvent mixture. When time was increased from 60 min to 120 min, a decrease in the lipid yield was observed for both species. The optimal temperature for lipid extraction for both species was determined to be 25 °C. A solid-to-solvent ratio of 1:20 for *Spirulina platensis* and a solid-to-solvent ratio of 1:50 for *Chlorella* sp. were the most efficient, and methanol was added as a co-solvent to improve the oil yield.

*Chlorella* sp. exhibited a significantly higher lipid content than *Spirulina platensis*.

However, lipids from both species have a high nutritional quality, with balanced ratios of saturated, monounsaturated, and polyunsaturated fatty acids, and are suitable for human consumption as a source of essential fatty acids and antioxidants. *Spirulina platensis* is more suitable as a food alternative, while *Chlorella* sp. is better suited as a source for biodiesel production, having a slight advantage due to its elevated lipid content.

Total SFA, MUFA, PUFA, and unsaturated fatty acids (UFA) were quantified in both microalgae species. *Chlorella* sp. has a higher omega-3 content, while *Spirulina platensis* has a higher omega-6 content. EPA + DHA content in *Spirulina platensis* (1.16) is higher than in *Chlorella* sp. (0.44), making it a better source of long-chain omega-3. The health promoting index is lower for *Chlorella* sp. than for *Spirulina platensis*. *Spirulina platensis* is a preferred food alternative, while *Chlorella* sp. is identified as a potential source for biodiesel production.

This study contributes to the promotion of microalgae biomass utilization for a variety of purposes, ensures that resources are used efficiently, and mitigates energy and solvent consumption. This aligns with the principles of circular economy, which emphasizes the efficient use of resources, the valorization of waste, and the development of knowledge on sustainable products with diverse applications. The results emphasized the potential of *Spirulina platensis* and *Chlorella* sp. as food or feed ingredients, as well as a potential source for applications in cosmetics, pharmaceuticals, and biofuels.

The study also optimizes processes to reduce environmental impact and enhances the use of microalgae lipids as a renewable resource, reducing the reliance on fossil fuels. The limitation of the present study was that this study was focused only on two microalgae species and one extraction method. Functional and nutritional analysis is also lacking, and further studies are needed to evaluate oxidative stability, bioavailability, and functional properties. Further research is needed to explore the potential of green solvents in enhancing the extraction efficiency and further reducing the environmental impact. Additional research to develop more effective methods for removing contaminants from microalgae biomass could facilitate the assurance of the safety and quality of the extracted lipids for food and pharmaceutical applications.

## Figures and Tables

**Table 1:** Composition of *Spirulina platensis* and *Chlorella* sp.

Parameter	*Chlorella* sp.	*Spirulina platensis*
Moisture (%)	0.7 ± 0.03 ^b^	1.1 ± 0.1 ^a^
Carbohydrates (%)	10.2 ± 1.01 ^b^	19.4 ± 1.13 ^a^
Lipid content (% dry wt)	17.4 ± 1.2 ^a^	7.2 ± 0.62 ^b^
Ash (%)	7.2 ± 0.52 ^a^	6.5 ± 0.41 ^a^
Protein (%)	59.3 ± 3.2 ^a^	65.6 ± 2.5 ^a^
Nitrogen (%)	9.5 ± 0.52 ^a^	10.5 ± 1.01 ^a^
Calorific value (kJ/g)	21.7 ± 1.81 ^a^	20.1 ± 1.8 ^a^
Cellulose (%)	13.2 ± 1.1 ^a^	15.4 ± 1.1 ^a^
Hemicellulose (%)	3.2 ± 0.20 ^a^	1.7 ± 0.15 ^b^

Note: The values are expressed as mean plus the standard deviation of three replicates. The letters ^a^ and ^b^ specify statistically significant differences at *p* < 0.05.

**Table 2:** Volatile organic compounds, chemical formula, chemical group, odor descriptor, and content (%) identified using HS-GC-MS for *Chlorella* sp. and *Spirulina platensis* microalgae biomass (data represent mean ± standard deviation, *n* = 3).

VOC	Molecular Formula	*Chlorella* sp.	*Spirulina platensis*	t_R_ (min)	Odor Descriptors *
Amines					
2-Propyn-1-amine	C_3_H_5_N	0.12 ± 0.01 ^a^	nd	4.689	Pungent, slightly fishy
N-Methylallylamine	C_4_H_9_N	nd	0.11 ± 0.01 ^a^	8.085	Strong, fishy, ammonia-like
3,4-Pyridinediamine	C_6_H_6_N_2_	0.05 ± 0.002 ^a^	nd	8.917	Amine-like, medicinal
2-Propen-1-amine	C_3_H_7_N	0.20 ± 0.01 ^b^	0.31 ± 0.02 ^a^	16.718	Strong, fishy
Ethylmethylamine	C_3_H_9_N	nd	0.59 ± 0.04 ^a^	18.450	Fishy, ammonia-like
N,N-2-trimethylpyridin-4-amine	C_6_H_11_N_2_	nd	3.50 ± 0.18 ^a^	20.49	Pungent, fishy
2-Methyl-2-propen-1-amine	C_5_H_11_N	0.36 ± 0.02 ^a^	0.35 ± 002 ^a^	25.112	Strong, amine-like odor
1H-Tetrazol-5-amine	C_2_H_3_N_3_	2.68 ± 0.02 ^a^	nd	40.15	Not found
5H-Tetrazol-5-amine	C_2_H_3_N_3_	1.01 ± 0.10 ^a^	0.41 ± 0.03 ^b^	17.537	Slightly pungent, medicinal
Aldehydes					
2-Methyl-2-butenal	C_5_H_10_O	0.33 ± 0.02 ^a^	nd	3.919	Pungent, strong, fruity
Hexanal	C_6_H_12_O	9.48 ± 0.61 ^b^	19.76 ± 1.52 ^a^	5.765	Green, grassy
4-Methylbenzaldehyde	C_6_H_7_NO	0.11 ± 0.01 ^a^	nd	21.384	Sweet, almond-like
Alcohols					
cis-(3,3,5)-trimethylcyclohexanol	C_9_H_18_O	nd	12.43 ± 0.98 ^a^	8.917	Woody, camphoraceous
6-Methyl-1-heptanol	C_8_H_18_O	nd	5.04 ± 0.21 ^a^	15.41	Floral, fruity, citrus
3,5-Dimethylcyclohexanol	C_8_H_14_O	5.13 ± 0.31 ^a^	nd	21.628	Sweet, floral
Ketones					
2,2,6-Trimethylcyclohexanone	C_9_H_16_O	nd	11.32 ± 1.1 ^a^	18.797	Sweet, woody
6-Methyl-2-azabicyclo[2.2.0]hex-5-en-3-one	C_7_H_11_NO	0.29 ± 0.01 ^a^	nd	23.123	Earthy
Acetone oxime	C_3_H_7_NO	0.62 ± 0.03 ^a^	nd	29.253	Slightly sweet, pungent
Hydrocarbon					
4,4-Dimethylcyclopentene	C_8_H_14_	7.24 ± 0.5 ^a^	nd	2.987	Slightly sweet, petroleum-like
Methoxyethylene	C_3_H_6_O	nd	1.85 ± 0.011 ^a^	3.000	Sweet, ether-like flavor
2-methyl-1,5-hexadien-3-yne	C_7_H_8_	1.02 ± 0.1 ^b^	2.18 ± 0.2 ^a^	4.463	Slightly sweet
1,2-Dodecane oxide	C_12_H_26_O	nd	6.33 ± 0.31 ^a^	21.622	Waxy, fatty, slightly floral
4-Ethylguaiacol	C_9_H_12_O_2_	nd	0.15 ± 0.01 ^a^	25.769	Sweet, smoky, spicy
Heterocycle					
6-Azaspiro[2.5]octa-4,7-diene-6-carboxylic acid	C_8_H_13_NO_2_	3.26 ± 0.18 ^a^	nd	6.640	Pungent
1,2,3,6-Tetrahydropyridine	C_5_H_9_N	nd	0.94 ± 0.06 ^a^	17.181	Musty, strong
Azetidine	C_3_H_7_N	2.59 ± 0.18 ^a^	nd	18.807	Slightly pungent
5-formamidopyrimidine	C_5_H_5_N_3_O	nd	0.52 ± 0.02 ^a^	19.357	Odorless
9-Aza-10-boradecalin	C_10_H_14_BN	7.82 ± 0.51 ^a^	nd	20.759	Not found
1-Oxaspiro[2.5]oct-5-ene, 8,8-dimethyl-4-methylene	C_12_H_14_O	nd	7.47 ± 0.42 ^a^	21.378	Slightly sweet, woody, herbal
Tetrahydropyridine	C_6_H_11_N	3.54 ± 0.21 ^a^	nd	22.835	Amine-like odor
Acids					
Aminooxyacetic acid	C_4_H_9_NO_3_	0.11 ± 0.01 ^a^	nd	9.630	Slightly sweet, amine-like
3-Chloropropionamide	C_3_H_6_ClNO	0.40 ± 0.02 ^a^	nd	24.90	Odorless
Ether					
Propylene oxide	C_3_H_6_O	0.09 ± 0.004 ^a^	nd	11.557	Slightly sweet, ether-like
Furans					
Furan	C_6_H_9_N	0.48 ± 0.02 ^a^	nd	16.211	Sweet, medicinal
2-Pentyl-furan	C_9_H_14_O	16.33 ± 1.2 ^a^	6.08 ± 0.30 ^b^	17.018	Pleasant, slightly sweet, nutty
Nitrogenous compounds					
N-methylaziridine	C_3_H_7_N	13.13 ± 0.1 ^a^	nd	2.768	Strong, pungent, amine-like
2,4-Hexadienenitrile	C_3_H_6_O	0.15 ± 0.01 ^a^	nd	16.155	Pungent, sharp, and acrid
3-Fluoro-2-propynenitrile	C_3_HFN	nd	0.13 ± 0.01 ^a^	23.254	Not found
Cyclopentaneacetonitrile	C_6_H_9_N	0.10 ± 0.01 ^a^	nd	7.923	Sweet, almonds
Sulfur compounds					
Methanesulfonyl fluoride	CH_3_SO_2_F	0.45 ± 0.03 ^a^	nd	19.758	Pungent, acrid
Esters					
Propyl cyanate	C_4_H_9_NO	8.57 ± 0.41 ^a^	nd	16.905	Pungent
Methyl sulfocyanate	CH_3_SCN	7.25 ± 0.53 ^a^	nd	17.13	Acrid, mustard-like odor, pungent

Values indicated with letters ^a^ and ^b^ were significantly different from each other at *p* ≤ 0.05 levels, nd—not determined; t_R_: retention time; * odor description attributed to [28].

**Table 3:** Fatty acids composition of *Chlorella* sp. and *Spirulina platensis* extracted lipids (%) (data represent mean ± standard deviation, *n* = 3).

Fatty Acids	Formula	*Chlorella* sp.	*Spirulina platensis*
Capric acid	C10:0	0.12 ± 0.01 ^a^	0.08 ± 0.02 ^b^
Undecanoic acid	C11:0	0.10 ± 0.01 ^b^	5.32 ± 0.21 ^a^
Myristic acid	C14:0	0.81 ± 002 ^a^	0.28 ± 0.02 ^b^
Myristoleic	C14:1(n-5)	nd	0.22 ± 0.01 ^a^
Pentadecanoic	C15:0	0.29 ± 0.01 ^a^	nd
Palmitic acid	C16:0	26.53 ± 1.8 ^b^	42.85 ± 2.3 ^a^
Palmitoleic acid	C16:1(n-7)	1.26 ± 0.12 ^b^	3.16 ± 0.18 ^a^
Margaric acid	C17:0	2.80 ± 0.2 ^a^	0.01 ± 0.001 ^b^
Heptadecenoic acid	C17:1(n-7)	nd	0.77 ± 0.03 ^a^
Stearic acid	C18:0	3.42 ± 0.2 ^a^	1.00 ± 0.11 ^b^
Oleic acid	C18:1(c+t)(n-9)	21.90 ± 1.8 ^a^	3.53 ± 0.21 ^b^
Linoleic acid	C18:2(c+t)(n-6)	25.32 ± 1.5 ^a^	14.43 ± 1.1 ^b^
γ-linolenic acid	C18:3(n-6)	0.14 ± 0.01 ^b^	22.45 ± 2.1 ^a^
α-Linolenic acid	C18:3(n-3)	10.23 ± 1.0 ^a^	0.47 ± 0.02 ^b^
Arachidic acid	C20:0	1.85 ± 0.1 ^a^	nd
Gondoic acid/*cis*-11-Eicosaenoic Acid	C20:1(n-9)	nd	0.72 ± 0.04 ^a^
*cis*-11,14-Eicosadienoic Acid	C20:2(n-6)	4.46 ± 0.3 ^a^	0.80 ± 0.03 ^b^
Heneicosylic acid	C21:0	nd	0.58 ± 0.02 ^a^
Eicosatrienoic acid	C20:3(n-3)	0.46 ± 002 ^b^	1.31 ± 0.09 ^a^
Eicosapentaenoic acid	C20:5(n-3)	0.02 ± 0.001 ^b^	1.16 ± 0.08 ^a^
Erucic acid	C22:1(n-9)	nd	0.12 ± 0.01 ^a^
Docosahexaenoic acid	C22:6(n-3)	0.42 ± 0.02 ^a^	nd
Free fatty acids (mg KOH/g)	FFAs	1.4 ± 0.1 ^a^	0.52 ± 0.02 ^b^
∑SFA		35.9 ± 2.1 ^b^	50.1 ± 2.2 ^a^
∑MUFA		23.2 ± 1.5 ^a^	8.5 ± 0.61 ^b^
∑PUFA		41.0 ± 1.8 ^a^	40.6 ± 2.3 ^a^
∑UFA		64.2 ± 2.2 ^a^	49.2 ± 2.8 ^b^
Omega-6		30.3 ± 2.6 ^b^	37.7 ± 2.6 ^a^
Omega-3		10.7 ± 0.9 ^a^	2.9 ± 0.17 ^b^

Note: Values indicated with letters ^a^ and ^b^ were significantly different from each other at *p* ≤ 0.05 levels, nd—not determined.

**Table 4:** Lipid nutritional indices calculated from FAMEs content for both microalgae.

Lipid Nutritional Indices	*Chlorella* sp.	*Spirulina platensis*
MUFA/SFA	0.64	0.17
PUFA/SFA	1.14	0.81
Omega-6 (%)	30.3	37.7
Omega-3 (%)	10.7	2.9
Omega-6/omega-3	2.83	13.0
Nutritive value index (NVI)	20.10	1.43
Atherogenicity index (AI)	0.46	0.89
Thrombogenicity index (TI)	0.22	0.28
Hypocholesterolemic/hypercholesterolemic ratio (h/H)	2.3	1.0
Health promoting index (HPI)	46.4	86.7
Linoleic acid/linolenic acid	0.014	47.52
LA/ALA (linoleic acid/α-linoleic acid ratio)	2.5	30.5
EPA + DHA (%)	0.44	1.16

**Table 5:** Calculation formula for lipid nutritional indices.

Index	Calculation Formulas
NVI	$\frac{\left(C18:0+C18:1\right)}{C16:1}$
AI	$\frac{\left[C12:0+\left(4\times C14:0\right)+C16:0\right]}{\sum UFA}$
TI	$\frac{\left(C14:0+C16:0+C18:0\right)}{[(0.5\times \sum UFA)+(0.5\times \sum PUFA\;n-6)+(3\times \sum PUFA\;n-6)+(\sum PUFA\;n-3/\sum PUFA\;n-6)]}$
h/H	$\frac{(cis-C18:1+\sum PUFA)}{\left(C12:0+C14:0+C16:0\right)}$
HPI	$\frac{\sum UFA}{\left[C12:0+(4\times C14:0)+C16:0\right]}$
EPA + DHA	C22:6(n-3) + C20:5(n-3)
LA/ALA	$\frac{C18:2(n-6)\;}{C18:3(n-3)}$

## Data Availability

Data is contained within the article.
